# Biosensor Based on Graphene Directly Grown by MW-PECVD for Detection of COVID-19 Spike (S) Protein and Its Entry Receptor ACE2

**DOI:** 10.3390/nano13162373

**Published:** 2023-08-18

**Authors:** Šarunas Meškinis, Rimantas Gudaitis, Andrius Vasiliauskas, Asta Guobienė, Šarūnas Jankauskas, Voitech Stankevič, Skirmantas Keršulis, Arūnas Stirkė, Eivydas Andriukonis, Wanessa Melo, Vilius Vertelis, Nerija Žurauskienė

**Affiliations:** 1 Institute of Materials Science, Kaunas University of Technology, K. Baršausko St. 59, LT-51423 Kaunas, Lithuania; rimantas.gudaitis@ktu.lt (R.G.); andrius.vasiliauskas@ktu.lt (A.V.); asta.guobiene@ktu.lt (A.G.); sarunas.jankauskas@ktu.lt (Š.J.); 2 Department of Functional Materials and Electronics, Center for Physical Sciences and Technology, Saulėtekio Ave. 3, LT-10257 Vilnius, Lithuania; voitech.stankevic@ftmc.lt (V.S.); skirmantas.kersulis@ftmc.lt (S.K.); arunas.stirke@ftmc.lt (A.S.); eivydas.andriukonis@ftmc.lt (E.A.); wanessa.melo@ftmc.lt (W.M.); vilius.vertelis@ftmc.lt (V.V.); nerija.zurauskiene@ftmc.lt (N.Ž.)

**Keywords:** graphene, direct synthesis, PECVD, field-effect transistor-based biosensor, COVID-19, SARS-CoV-2 spike protein, receptor ACE2, charge neutrality point, shift of Dirac voltage

## Abstract

Biosensors based on graphene field-effect transistors (G-FET) for detecting COVID-19 spike S protein and its receptor ACE2 were reported. The graphene, directly synthesized on SiO_2_/Si substrate by microwave plasma-enhanced chemical vapor deposition (MW-PECVD), was used for FET biosensor fabrication. The commercial graphene, CVD-grown on a copper substrate and subsequently transferred onto a glass substrate, was applied for comparison purposes. The graphene structure and surface morphology were studied by Raman scattering spectroscopy and atomic force microscope. Graphene surfaces were functionalized by an aromatic molecule PBASE (1-pyrenebutanoic acid succinimidyl ester), and subsequent immobilization of the receptor angiotensin-converting enzyme 2 (ACE2) was performed. A microfluidic system was developed, and transfer curves of liquid-gated FET were measured after each graphene surface modification procedure to investigate ACE2 immobilization by varying its concentration and subsequent spike S protein detection. The directly synthesized graphene FET sensitivity to the receptor ACE2, evaluated in terms of the Dirac voltage shift, exceeded the sensitivity of the transferred commercial graphene-based FET. The concentration of the spike S protein was detected in the range of 10 ag/mL up to 10 μg/mL by using a developed microfluidic system and measuring the transfer characteristics of the liquid-gated G-FETs. It was found that the shift of the Dirac voltage depends on the spike S concentration and was 27 mV with saturation at 10 pg/mL for directly synthesized G-FET biosensor, while for transferred G-FET, the maximal shift of 70 mV was obtained at 10 μg/mL with a tendency of saturation at 10 ng/mL. The detection limit as low as 10 ag/mL was achieved for both G-FETs. The sensitivity of the biosensors at spike S concentration of 10 pg/mL measured as relative current change at a constant gate voltage corresponding to the highest transconductance of the G-FETs was found at 5.6% and 8.8% for directly synthesized and transferred graphene biosensors, respectively. Thus, MW-PECVD-synthesized graphene-based biosensor demonstrating high sensitivity and low detection limit has excellent potential for applications in COVID-19 diagnostics.

## 1. Introduction

COVID-19, also known as the coronavirus disease 2019, is a recently emerged infectious disease that affects humans and causes severe respiratory distress. It was first identified in 2019 as a novel coronavirus, referred to as 2019-nCov, which was found in the bronchoalveolar lavage fluid of a patient. Subsequently, the virus was renamed severe acute respiratory syndrome coronavirus 2 (SARS-CoV-2) by the International Committee on Taxonomy of Viruses [1]. Due to its rapid spread among individuals, the World Health Organization (WHO) declared the COVID-19 outbreak as a pandemic on 12 March 2020. As of July 2023, there have been more than 767,000,000 confirmed cases of COVID-19 worldwide, resulting in almost 7 million deaths, according to the World Health Organization’s official website (https://covid19.who.int, accessed on 13 July 2023). Thus, these data emphasize that the virus is still present and more research is needed. Although the specific mechanism by which SARS-CoV-2 causes the disease is not fully understood, recent research has indicated that SARS-CoV-2 utilizes angiotensin-converting enzyme II (ACE2) as a receptor to enter host cells. It is noteworthy that ACE2 also is a known cellular receptor for SARS-CoV [2]. In animal cells, SARS-CoV-2, and ACE2 are found in close proximity, and the virus’s spike (S) protein exhibits a strong binding affinity for ACE2 [3,4].

The growing prevalence of diseases and their associated risks necessitates the exploration of novel sensor types for detection purposes. The recent encounter with the COVID-19 pandemic highlighted the crucial significance of virus detection methods, particularly due to the virus’s rapid mutation and emergence of different variants.

Among the many diagnostic methods currently available, field-effect transistor (FET)-based biosensor devices can be used for virus detection [5,6,7,8]. These devices offer several advantages due to their small size and sensitivity, including the ability to make highly sensitive and instantaneous measurements using small amounts of analytes. Furthermore, FET-based biosensors are considered one of the perspective types of sensors to be potentially valuable for point-of-care testing, particularly for on-site detection, with the possibility to integrate on a chip using complementary metal oxide semiconductor (CMOS) technology [5,6,7,8,9]. Two-dimensional (2D) nanomaterials offer many unique possibilities for FET-based biosensors due to their atomically thin nature and extra-large surface-to-volume ratio [8]. Graphene, a two-dimensional carbon allotrope composed of a single layer of carbon atoms arranged in a honeycomb lattice, was the first isolated 2D nanomaterial to be studied [10]. It has garnered tremendous attention in the scientific community due to its extraordinary electronic and mechanical properties. Notably, ultra-high charge carrier mobility of 350,000 cm^2^V^−1^s^−1^ [11], Young’s modulus of 1 TPa [12], ease tunable electrical properties [8,13,14,15], large surface area and ability to sense molecules [8,13,16,17] offer significant advantages for applications. Remarkably, the large surface-to-volume ratio of the graphene provides a lot of the exposed atoms available for functional group attachment, resulting in significantly enhanced surface reactivity. Combined with easily tunable electrical properties, it opens up new possibilities for chemical sensing [18]. Therefore, graphene became a viable option for biosensing applications [8,13,16,19,20].

The operation of graphene field-effect transistors (G-FETs) designed as bioanalytical sensors are based on the change of the electrical conductance (resistance) or electrical current flowing through the sensor induced by changes in the environment and the interaction with bioanalytes (DNA sequences, bacteria, viruses, drugs, etc.) [21,22,23]. Due to the atomically thin graphene layer, the accumulation of bioanalytes on the surface of functionalized graphene induces a significant response of the G-FET. It thus ensures high sensitivity and increased detection limit to less than femtomolar concentrations [8,24]. One of the most investigated characteristics for the measure of biomolecules is a transfer characteristic of the FET (dependence of drain (D)—source (S) current, *I*_DS_, on the gate voltage *V*_GS_ while keeping the bias voltage *V*_DS_ constant). Such dependences have a minimum at a specific gate voltage *V*_GS_ = *V*_D_ called Dirac point or charge neutrality point (CNP). This transfer characteristic’s left and right branches moving away from the CNP are called the p-branch and n-branch, respectively. They are related to the increasing density of positive charge carriers (holes) or negative charge carriers (electrons). Due to the zero-bandgap structure of graphene, carriers (electrons and holes) can be changed to each other at the Dirac point by increasing ambipolar gate voltage, which results in the formation of bipolar transfer characteristics of G-FET [25]. At the CNP, the current reaches a minimum indicating an equal density of holes and electrons. One expects the *V*_D_ to be close to *V*_GS_ = 0 V for good quality (e.g., few defects and adsorbants) intrinsic graphene at low bias voltage *V*_DS_. However, in real cases, it is shifted to positive gate voltages if graphene is p-doped or negative voltages if it is n-doped. The doping depends on various factors, such as substrate, metal of electrodes, defects and wrinkles of transferred graphene, nonhomogeneous distribution of charged species forming electron and hole paddles, etc. [8,26]. It is worth mentioning that a significant shift of the *V*_D_ from its initial value is obtained due to the interaction of the functionalized graphene surface with an analyte and could be a measure of the concentration of the analyte molecules. Therefore, preparing good structural quality graphene layers and process reproducibility is essential for developing highly sensitive G-FET biosensors.

The main methods used for graphene synthesis today are either exfoliation or chemical vapor deposition (CVD) on copper or nickel films with an additional transfer of graphene film to a required substrate. The exfoliation yields pristine graphene samples. However, the reproducibility of this process is complicated [27,28]. Combining the chemical vapor deposition on the catalytic metal foil with subsequent transfer to the targeted substrate produces much larger graphene samples. However, this prolonged process still needs to be improved for industrial applications [29,30]. It can even produce cracks and tears in the transferred graphene sheets [31], not to mention different organic adsorbates that accumulate on the graphene surface during the transfer [32]. The alternative is direct graphene synthesis on dielectric or semiconducting substrates via plasma-enhanced chemical vapor deposition (PECVD). This deposition method eliminates graphene transfer-related problems and keeps most of the pristine graphene properties required for biosensing applications intact [27,33]. In this case, control of the graphene flakes’ orientation and growth of both planar graphene and vertical graphene nanowalls is possible [34,35]. However, direct graphene synthesis and its application for biosensor fabrication are much less studied than exfoliated or CVD-grown and transferred graphene. In some studies, graphene directly synthesized on dielectric substrates without using any catalyst was already successfully used as a FET channel [36,37,38,39,40,41,42]. However, only in [41], an initial study on the use of the directly synthesized graphene-based FET (G-FET) as a biosensor for adenosine tri-phosphate detection was presented. Despite that, in the recent review on graphene-based FET biosensors [8], it was pointed out that direct synthesis of the graphene on an insulating substrate is a promising technology for manufacturing G-FET-based biosensors.

This study presents the findings of examining graphene directly grown on the SiO_2_ substrate through the microwave plasma-enhanced chemical vapor deposition (MW-PECVD) technique for its suitability in biosensors designed to detect the spike (S) protein of COVID-19. The results are compared with biosensors fabricated using commercially grown graphene on a copper substrate, which was subsequently wet transferred to a glass substrate.

## 2. Materials and Methods

### 2.1. Synthesis of MW-PECVD Graphene

Graphene samples were produced using a microwave plasma-enhanced chemical vapor deposition system, Cyrannus (Innovative Plasma Systems (Iplas) GmbH, Troisdorf, Germany). The Si(100) wafer coated by 300 nm thickness silicon dioxide (SiO_2_) film using thermal oxidation was used as a substrate. During the fabrication process of the sensor, graphene was grown on a SiO_2_/Si substrate with dimensions of 15 × 15 mm^2^. The graphene synthesis conditions were based on our previous studies [35,43,44] and are outlined in Table 1.

Afterward, the graphene was patterned to obtain active area dimensions of 3 × 3 mm^2^. At first, copper film was deposited through mask using electron beam evaporation. Then, unprotected graphene was etched out using oxygen plasma. Finally, the copper mask was removed by wet chemical etching. To create electrical contact, two Ag electrodes (source and drain) were deposited using unbalanced DC magnetron sputtering. The thickness of the silver film was 250 nm. The dimensions of the electrodes were 7 × 3 mm^2^, and the distance between them was 1.5 mm. Thus, the metal electrodes covered two 3 × 0.75 mm^2^ areas of the graphene surface, creating a low contact resistance between the graphene and the electrodes.

### 2.2. Preparation of Graphene by Wet Transfer Procedure

For comparison, a sensing element with graphene transferred from Cu foil was also used for biosensor fabrication. During the fabrication process, the graphene grown on a Cu foil (Graphenea Semiconductor SLU) was transferred onto a 1 mm thick glass substrate with already deposited Ag contacts using a wet chemical etching procedure. The Ag electrodes, with a Cr sublayer, were thermally deposited on the top of the glass substrate and post-annealed at 450 °C for 1 h in an Ar atmosphere for good adhesion with the substrate. The electrodes had the same configuration as in the previous case (MW-PECVD graphene). During the transfer procedure, the Cu foil with graphene was cut into pieces of 3 × 3 mm^2^; then, the Cu was chemically dissolved using an ammonium persulfate solution from the bottom of the Cu/Graphene/PMMA structure. As a result, the single-layer graphene (SLG) with a PMMA flake was floating on the surface of the etching solution. Subsequently, rinsing with deionized water was performed, and the floating flake was captured and transferred onto the glass substrate (15 mm × 15 mm) with the pre-existing Ag contacts. The PMMA layer was removed using acetone and isopropanol just before the functionalization of the graphene film. To ensure good contact between the electrodes and graphene, an additional deposition of Ag electrodes with the same configuration was applied on the top of the graphene. As a result, at the point of contact, the electrodes covered both sides of the graphene layer.

### 2.3. Characterization of Graphene Sensing Elements

Transferred and directly grown graphene samples were characterized using Raman spectroscopy. Raman spectroscopy measurements of graphene were carried out using the InVia Raman microscope (Renishaw, Wotton-under-Edge, UK) equipped with a thermoelectrically cooled (–70 °C) CCD detector and 532 nm laser radiation source. The step size was 30 × 35 µm in x- and y-directions; each map consisted of 50 measurements with 20 s acquisition time at 0.23 mW laser power focused on a sample using 50×/0.75 NA objective lens (Leica). 2D and G intensity ratio (2D/G) was plotted using Wire 5.5 (Renishaw, Wotton-under-Edge, UK).

**Figure 1:**
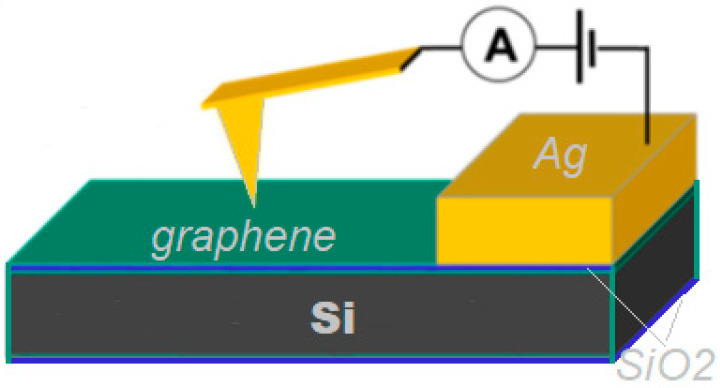
The schematic of C-AFM used for measurements of the graphene surface conductivity.

The directly synthesized graphene surface conductivity and morphology of graphene sheets were analyzed by atomic force microscopy (NanoWizard^®^3, JPK Instruments, Bruker Nano GmbH, Berlin, Germany). The morphology and phase images were collected using an ACTA (Applied NanoStructures, Inc., Mountain View, CA, USA) probe operating in tapping mode. The probe tip radius of curvature was 6 nm. More details regarding these measurements can be found in [35,45]. To investigate the surface electrical conductance, the special Ag electrodes were formed over the graphene layer (see Figure 1). The conductivity was measured using contact-mode conductive atomic force microscopy (C-AFM) with a metal-coated tip ANSCM-PT (AppNano, Mountain View, CA, USA) silicon probe with a thin layer of Pt/Ir coating (thickness (nm) −25 ± 5) on both reflex and tip sides of the probe. ANSCM probes with a 1.6 spring constant are ideal for use in C-AFM mode. Tip Shape-Tetrahedral; Tip ROC (nm)—30, Height (μm), 14–16, Frequency (kHz)—61. The electrical current was measured as a function of the applied bias voltage (−10–10 mV). All the measurements were performed at room temperature in the air.

### 2.4. Preparation of Graphene Biosensor

**Figure 2:**
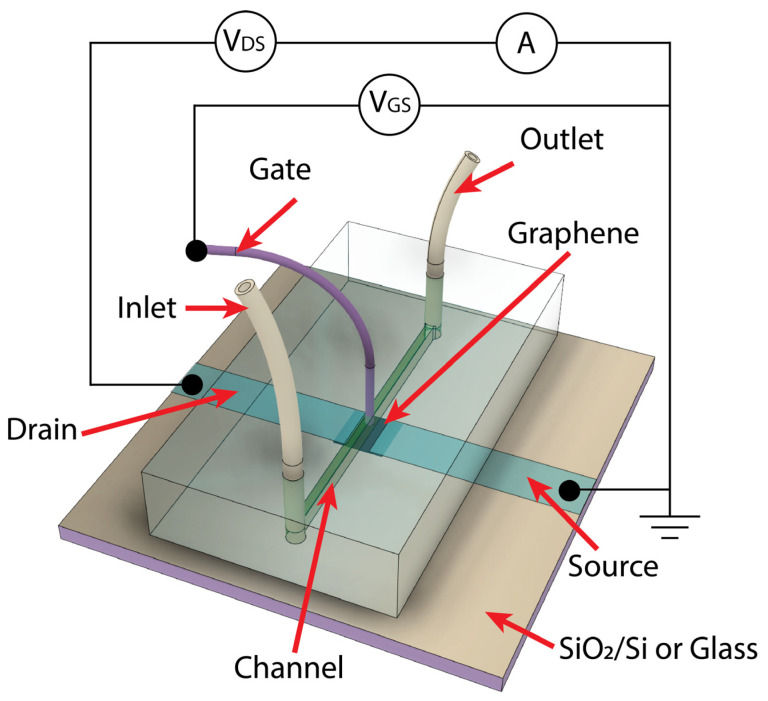
Graphene-sensing element with microfluidic system and measurement scheme.

In order to make a graphene-based sensor sensitive to the spike (S) protein of COVID-19, the surface of graphene was functionalized. For this purpose, the fabricated graphene-based elements underwent a soaking process in a solution of 1 mM 2.5-dioxopyrrolidin-1-yl 4-(pyrene-1-y) butanoate (PBASE) (Cayman Chemical, Ann Arbor, MI, USA) in methanol (VWR International, France) at room temperature for one hour. This procedure was performed based on a reference [46] with certain adjustments. After that, the graphene-based element was left overnight in the oven at 60 °C. The prepared PBASE/graphene samples were used in the microfluidic system in order to create a liquid-gated field effect transistor (FET) structure for the subsequent experiments. The microfluidic system (see Figure 2) was created by covering the graphene-based sensing element by specially prepared polydimethylsiloxane (PDMS) chips with microfluidic channels with a width of 0.5 mm and a length of 3 mm. So, only the graphene under the channel (0.5 mm × 3 mm) was exposed to the liquid analyte. The microfluidic channel was prepared using master mold casting technics. The cast PDMS (Sylgard^TM^ 184 Silicone Elastomer, DOW Europe, Germany) mold was cured at 60 °C overnight. Then, de-molded PDMS was punctured using a biopsy puncher to obtain inlets to which two flexible tubes were connected. The liquid gate was formed by inserting a stainless steel wire with a 200 μm diameter through the PDMS chips, positioning the end of the wire at the center of the channel directly over the graphene, and protruding from the channel to be submerged in the liquid by approximately 0.1 mm (0.4 mm away from the surface of graphene). A gate voltage was then connected to the opposite end of the wire. To ensure sealing between the elastomer and the surface of the graphene-based device, a custom 3D-printed holder was used (not shown in the figure). Moreover, this holder had a set of connectors for easy connecting of measurement devices.

First, the PBASE functionalized device was exposed to several concentrations (from 10 ag/mL to 10µg/mL) of the recombinant angiotensin-converting enzyme 2 (ACE2) (Baltymas UAB, Vilnius, Lithuania) protein prepared in 0.1× phosphate-buffered saline (PBS) (contains 0.0137 M of NaCl (VWR International, Villers-lès-Nancy, France), 0.00027 M of KCl (VWR International, Villers-lès-Nancy, France), 0.001 M of Na_2_HPO_4_: (Sigma Aldrich (St. Louis, MO, USA), and KH_2_PO_4_: 0.00018 mM (Merck, Darmstadt, Germany)) at least for 4 h at room temperature. The excess ACE2 was removed by rinsing 1 mL of PBS with Tween-20 (0.05%) and then with 2 mL of PBS only. Then, 1 mL of PBS with 0.1% concentration of Bovine Serum Albumin (BSA) (Sigma Aldrich (St. Louis, MO, USA) was injected into the microfluidic system to block ACE2 uncovered places. Finally, the experiments with a different recombinant SARS-CoV-2 Spike glycoprotein trimeric ectodomain (S) (Baltymas UAB, Vilnius, Lithuania) of protein concentrations ranging from 10 ag/mL to 10 µg/mL prepared in 0.1× PBS (chemical composition of PBS described above) were performed.

### 2.5. Electrical Measurements

The schematic of the measurement circuit for liquid-gated G-FET biosensors is shown in Figure 2. The electrical performance was evaluated using a YOKOGAWA GS610 source measurement unit from Japan and an ITECH IT6123 DC source meter from the US. The G-FET transfer characteristics, i.e., dependences of a drain (D)-source (S) current *I*_DS_ vs. gate voltage *V*_GS_ were measured while maintaining constant bias voltage *V*_DS_ of 50 mV. Prior to conducting transfer characteristic measurements, real-time (kinetics) resistance measurements of the graphene channel were carried out. Following each process of the functionalization of the graphene surface, a waiting period of 30 min was implemented. This duration proved adequate for relaxation processes to occur and for the resistance saturation value to be attained.

During the experiments, the voltage of the Dirac point *V*_D_ was determined as the gate voltage at a minimum current value in the *I*_DS_-*V*_GS_ characteristics of the liquid-gated G-FET. The voltage applied to the gate (*V*_GS_) was swept within the range of ±0.8 V.

## 3. Results and Discussion

### 3.1. Graphene Structure and Properties

**Figure 3:**
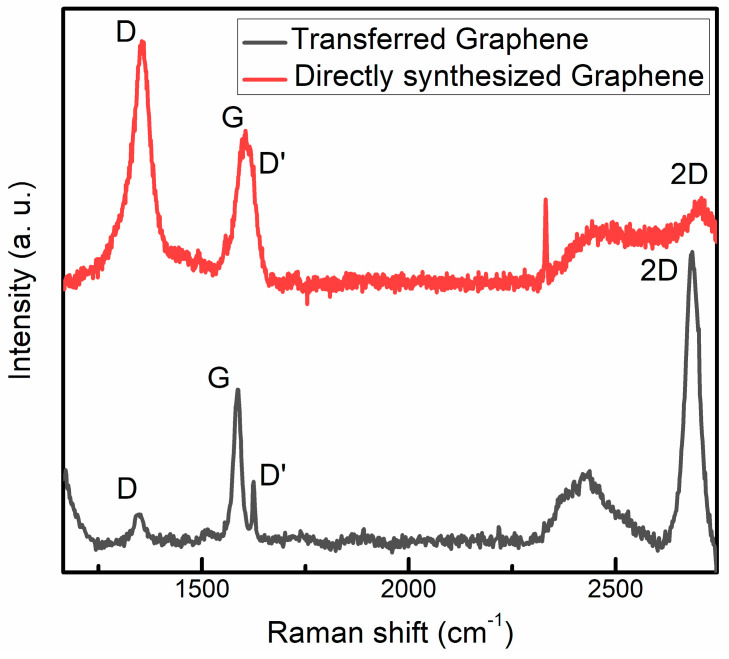
Typical Raman spectra of CVD grown and transferred graphene and graphene directly synthesized by MW−PECVD.

The Raman scattering spectra are presented in Figure 3. The spectrum of CVD-grown and transferred graphene is typical for pristine graphene [15,47,48]. Two prominent peaks, namely the G and 2D peaks, were observed. The G peak corresponds to lattice vibrations, while the 2D peak originates from second-order Raman scattering [47]. Notably, the average I_2D_/I_G_ ratio of CVD graphene was approximately 1.94 (Table 2), indicating that it is single-layer graphene [49]. Weak defect-related D and D’ peaks [47,50] can be seen, too. In the case of the transferred graphene, the I_D_/I_D’_ ratio was 0.55 (Table 2). Thus, the presence of the on-site defects induced by transfer-related adsorbants can be supposed [50]. The same main peaks were found in Raman scattering spectra of the graphene directly synthesized by MW-PECVD. However, in this case, the D peak was much stronger, resulting in a significantly larger I_D_/I_G_ ratio, indicating a much larger defect density. Another defect-related peak, D’ peak, was merged with the G peak and was seen as a shoulder. An analysis of the I_D_/I_D’_ ratio (Table 2) revealed the prevalence of the boundary defects in the directly synthesized graphene. That can be explained by the nanocrystalline nature of the graphene directly synthesized by MW-PECVD. In our case, the average nanocrystal size was below 10 nm. The average nanocrystal size (*L*_a_), estimated using the equation of the Tuinstra and Koenig *L*_a_ = 4.4/(I_D_/I_G_) nm [51,52], was ~3 nm. While the average nanocrystal size calculated using the equation proposed by P. Mallet-Ladeira et al. [53] *L*_a_ = (68-FWHM(G))/5.2 nm was ~5.8 nm. According to the [49], the I_2D_/I_G_ ratio of the directly synthesized graphene indicates the formation of the three-layer and four-layer graphene. G-band peak position (Pos(G)) of the Raman spectra of directly synthesized graphene was upshifted compared to the Raman spectra of transferred graphene, and FWHM(G) was much increased. Along with the increased I_D_/I_G_ ratio, it indicates increased defects density [54]. That can be explained by a large number of boundary defects due to the directly synthesized graphene nanocrystalline nature. The increase in the FWHM(2D) can indicate the presence of compressive stress in the directly synthesized graphene [55,56]. The upshift of the 2D peak position (Pos(2D)) and Pos(G) was in good accordance with this assumption [48,57,58,59]. The upshift of the Pos(2D) and Pos(G) can also be related to graphene doping [48,58,59]. However, FWHM(G) should decrease with graphene doping [54,55]. In contrast, we observed an opposite trend. Thus, if there was an effect of possible doping of graphene, it was overshadowed by the impact of defects.

**Figure 4:**
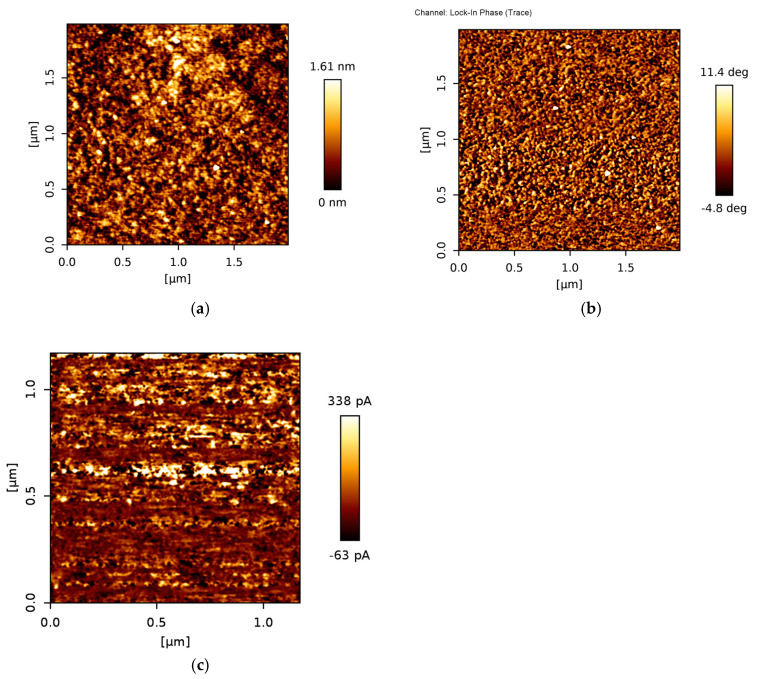
Directly synthesized graphene 2D morphology (**a**), phase (**b**) and conductivity (**c**) images.

The morphology and conductivity of the directly synthesized graphene were additionally studied by atomic force microscopy. The graphene consisted of the 20–25 nm size features (Figure 4). The height of those grains was 1–1.3 nm (Figure 4). Taking into account that graphene monolayer thickness is ~0.4 nm [60], the abovementioned feature height was in good accordance with the directly synthesized graphene layer number estimated by Raman scattering spectroscopy. The phase image was very similar to the 2D morphology image, confirming the observation of the graphene nanograins or nanoflakes. Considering average graphene nanocrystallite size evaluated using Raman scattering spectroscopy, in our case, 20–25 nm nanocrystalline graphene flakes consisted of the 3–6 nm average size nanocrystallites.

The conductivity image revealed the presence of very different conductivity areas (Figure 4c). This can be explained by the nanocrystalline nature of the directly synthesized graphene due to the much lower conductivity of the grain boundaries [61]. The average current measured by CAFM was 440 pA, and the maximum current was 1.6 nA.

### 3.2. Immobilization of G-FET Sensing Elements by Receptor ACE2

Before performing the experiments with a recombinant SARS-CoV-2 Spike (S), the ACE2 receptors were immobilized on PBASE/graphene. To enhance the immobilization of ACE2 on the PBASE, the variations in ACE2 concentration were examined.

**Figure 5:**
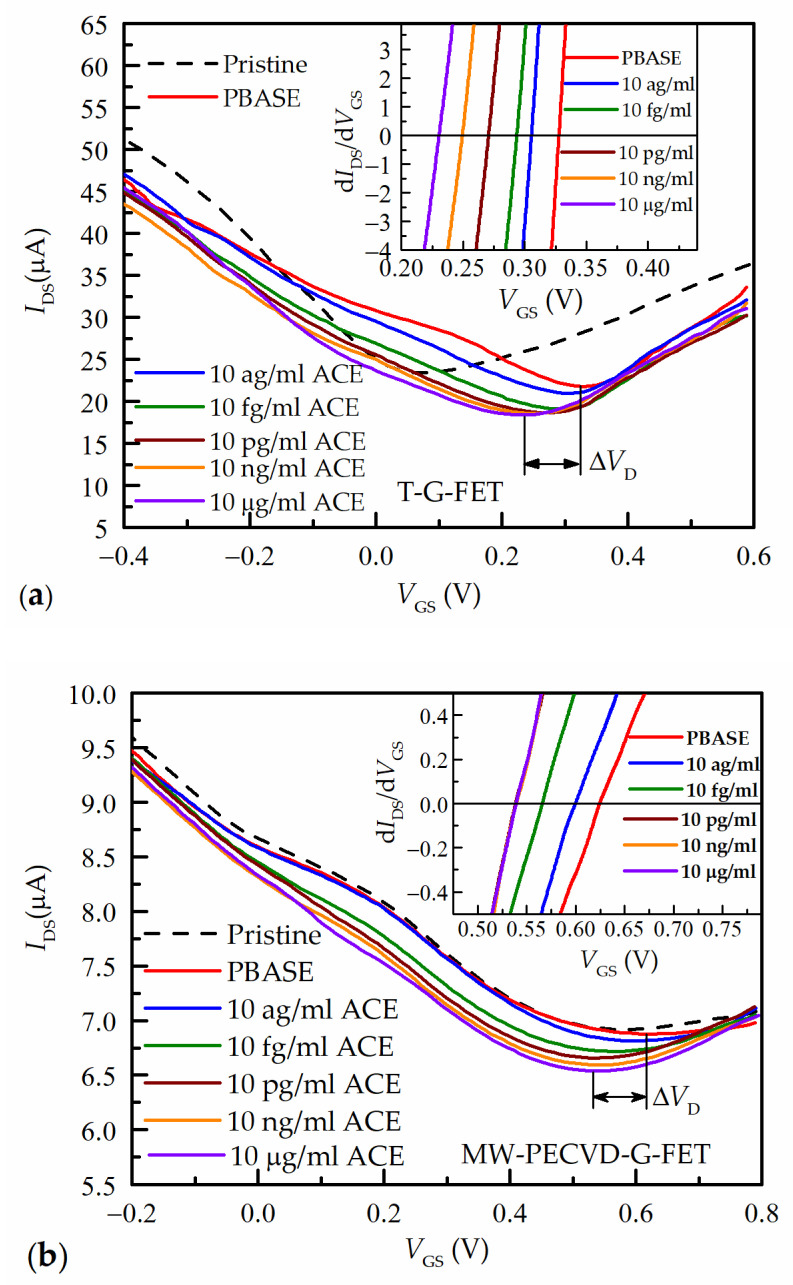
G-FET transfer characteristics at different ACE2 concentrations for T-G-FET (**a**) and MW-PECVD G-FET (**b**) sensors. Insets: the derivatives of transfer characteristics close to Dirac point at which derivative is equal to zero.

Figure 5 presents the characteristic transfer curves (*I*_DS_ vs. *V*_GS_) of both G-FETs for different concentrations of ACE2 in the PBS solution. All curves had the characteristic minimum, which corresponded to the Dirac point (charge neutrality point, CNP). For a more accurate determination of the position of the minimum, the derivatives of these curves are shown in the inset. The voltages at which d*I*_DS_/d*V*_GS_ = 0 corresponded to the Dirac point.

For comparison, the measurements of G-FET transfer characteristics of pristine graphene elements before the functionalization by PBASE are also shown (dashed black curve). In general, for detecting biomolecules, the key parameter of significance is the magnitude of the shift in the Dirac voltage. However, it should be noted that for graphene, the surface of which is functionalized, the primary transfer characteristic corresponding to pristine graphene (exhibiting a Dirac voltage closer to zero) is also very critical. This is because contaminants on the graphene surface not only affect its doping but also diminish carrier mobility and restrict surface functionalization [62]. One can see from the graphs in Figure 5a that for transferred graphene T-G-FET the Dirac voltage of pristine graphene is equal to *V*_D_ = 74 mV, meanwhile for MW-PECVD-G-FET (see Figure 5b), it is shifted up to *V*_D_ = 580 mV. In general case, the doping level of pristine graphene is influenced by various factors, which include the interaction of the interface between graphene and other materials (such as substrate, electrodes, and media) and the distribution of charged species or impurities within these materials [63]. The results obtained for pristine graphene in MW-PECVD-G-FET can be explained based on the results of previous works: a similar positive shift of the Dirac voltage was reported for graphene directly synthesized on the SiO_2_ film without the use of any dopants in [37,39,42]. However, no shift was found for graphene directly synthesized on the molten glass and afterward transferred onto the SiO_2_ film [64]. Therefore, substrate-induced p-type doping of the directly synthesized graphene can be supposed. It should be mentioned that substrate-induced graphene self-doping was reported in numerous studies [65,66,67,68,69,70,71]. Notably, p-type self-doping can be induced in the graphene transferred onto the SiO_2_ substrate due to the different surface treatments and residual charges created on the substrate [65]. It was found that the presence or absence of the graphene self-doping phenomena and graphene self-doping type (p-type or n-type) can be controlled by selecting the appropriate substrate [68]. The modeling revealed that the electronic structure of the graphene placed onto the SiO_2_ significantly depends on the interface geometry and surface polarity [69]. O-polar SiO_2_ surface with dangling bonds induces p-type doping in the graphene [69]. Using the Si-polar surface with dangling bonds results in the graphene n-type self-doping [69]. Thus, one can suppose that substrate treatment before graphene growth and/or the direct graphene synthesis process itself resulted in changes in the substrate surface or interface composition and the resultant transfer of the induced positive charge to the graphene.

Nevertheless, for transferred graphene T-G-FET, the quality of the fabrication process can also be associated with the level of doping, as it can indicate the amount of impurities present between graphene and the substrate after the transfer process [41,72]. Although during the transfer of graphene on the surface of the glass, the effort to reduce contaminants in the transfer process was made by chemical cleaning and treatment in oxygen plasma, the Dirac voltage of pristine graphene was shifted from 0 V: *V*_D_ = 74 mV.

As was mentioned before, the pristine graphene sensing elements were functionalized by PBASE. One can see that for transferred graphene T-G-FET (Figure 5a), a shift of minimum (*V*_D_) to higher gate voltages *V*_GS_ of the transfer curve after the covering of the graphene by PBASE was much larger in comparison to ME-PECVD-G-FET (Figure 5b): from 74 mV up to 327 mV (Δ*V*_D_ = 253 mV) and from 580 mV up to 624 mV (Δ*V*_D_ = 44 mV), respectively. The shift in the positive direction by PBASE can be explained by its p-doping effect through the charge transfer between the pyrene group and graphene [73]. This shift confirms the successive surface functionalization by the PBASE. However, for the MW-PECVD-G-FET, the small shift of the minimum indicates that a smaller area of the graphene channel was functionalized by PBASE. This can be caused by numerous grain boundary defects in the directly grown graphene, which is nano-crystalline with a large number of grain boundaries (see Figure 4). It should be noted that the defects and contaminations over the graphene surface induce electrical characteristics that vary from sample to sample and lead to the increase in the Dirac point of the pristine graphene. This is consistent with the result of Raman spectroscopy studies of the MW-PECVD-grown graphene on SiO_2_/Si substrates. Moreover, as was mentioned before, the Dirac point shifted only by several tens of millivolts after the functionalizing of the surface of the MW-PECVD graphene, indicating the smaller active area of graphene in this case.

For the immobilization of receptor ACE2 on the PBASE, the microfluidic channel was filled by ACE2, and transfer characteristics of both G-FETs were measured for each concentration. The concentration of ACE2 in the microfluidic system was increased step by step from the lowest (10 ag/mL) to the highest (10 mg/mL). The results of these measurements are shown in Figure 5. The immobilization of ACE2 caused the shift of *V*_D_ to the lower voltage depending on the concentration—the higher the concentration of ACE2, the larger shift of *V*_D_ takes place. The reason for the shift in the Dirac point in the negative direction is related to a negative charge induced by ACE2 on the PBASE/graphene surface.

**Figure 6:**
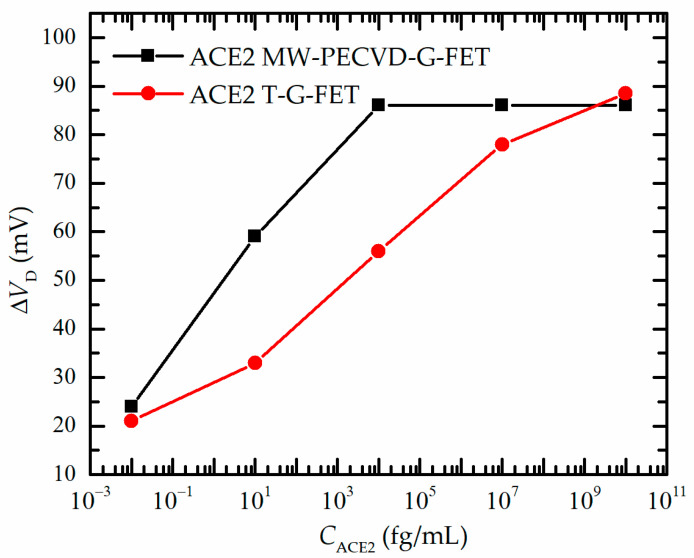
Change of *V*_D_ vs. ACE2 receptor concentrations for PECVD-G-FET and T-G-FET.

Figure 6 summarizes the data presented in Figure 5. As shown in Figure 6, the Dirac voltage shift (Δ*V*_D_) was observed as a response to ACE2 concentration *C*_ACE2_ for both G-FETs. First, MW-PECVD-G-FET showed quite good sensitivity at the lowest ACE2 concentrations. Even at 10 ag/mL (or 10^−2^ fg/mL) of ACE2 protein, it was possible to detect measurable signals. Then, at higher concentrations, it tended to saturate when it reached 10^4^ fg/mL, which corresponded to the maximal Δ*V*_D_ =86 mV. The saturation at lower concentration in comparison to transferred graphene (red curve) may be due to the specific features of the graphene layer nanostructure of MW-PECVD-G-FET described above: due to a large number of grain boundaries and small size nanocrystallites, the relative area of perfect graphene which could be functionalized was smaller in comparison to transferred graphene. Therefore, for the T-G-FET, the saturation of the Δ*V*_D_ value occurred at much higher concentrations. One can see that the shift of the Dirac voltage tended to saturate at 10^7^ fg/mL. However, in all cases, the Δ*V*_D_ of the MW-PECVD-G-FET was larger than the ΔV_D_ of the T-G-FET, revealing good functionalization possibilities of directly synthesized graphene.

### 3.3. Detection of SARS-CoV-2 Spike S Protein

To demonstrate and to compare G-FETs biosensing properties, recombinant SARS-CoV-2 spike (S) and recombinant angiotensin-converting enzyme 2 (ACE2) proteins were selected as a model. It is known that ACE2 is recognized as the entry receptor for SARS-CoV-2 [74]. This fact was explained through structural studies, which showed that SARS-CoV-2 spike (S) glycoproteins exhibit a stronger binding affinity to ACE2 [75,76]. Additionally, the overall structure of SARS-CoV-2 S closely resembles that of SARS-CoV S, with the spike receptor-binding domain (RBD) making contact with the extracellular region of ACE2 [77].

Based on the studies conducted on the immobilization of PBASE/graphene with the ACE2 receptor, we concluded that an optimal concentration of 10 µg/mL of ACE2 is required for T-G-FET, while an ACE2 concentration of 10 ng/mL could be sufficient for MW-PECVD-G-FET. Therefore, during the subsequent research, the concentration of 10 µg/mL of ACE2 was used for the preparation of both G-FET biosensors to ensure further comparison of obtained results.

In the next step, the surface of the graphene sensor was rinsed with 1 mL of PBS with Tween-20 and then with 2 mL of PBS to remove the excess ACE2 and to passivate the uncoated surface of the graphene. Subsequently, 1 mL of PBS with 0.1% Bovine Serum Albumin (BSA) (Sigma Aldrich, St. Louis, MO, USA) was injected into the microfluidic system to block any ACE2-uncovered areas. Following this, biosensors were prepared and utilized to examine the detection of the recombinant S protein by G-FETs.

**Figure 7:**
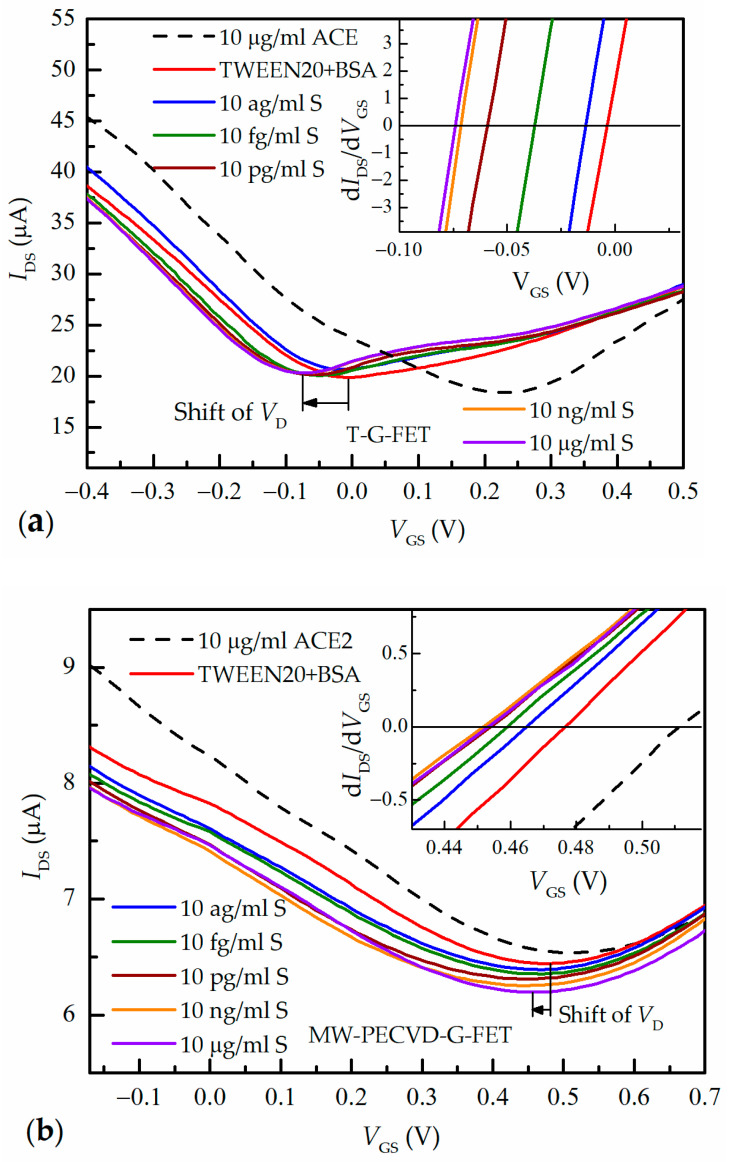
G-FET transfer characteristics at different spike S protein concentrations for T-G-FET sensors (**a**) and MW-PECVD G-FET (**b**). Insets: the derivatives of transfer characteristics close to the Dirac point at which the derivative is equal to zero.

Figure 7 presents the characteristic transfer curves (*I*_DS_ vs. *V*_GS_) of both G-FETs for different concentrations of S protein in a PBS solution. For comparison, the measurements of the G-FET transfer characteristics of graphene elements immobilized by ACE2 with a concentration of 10 µg/mL (black dashed line) and after rinsing with Tween-20 and subsequent treatment with BSA (red line) are shown.

It can be observed that the rinsing procedure induced significant changes in the position of the minimum on the transfer curves. This was particularly evident in the samples of transferred graphene T-G-FET (see Figure 7a), where the minimum (*V*_D_) shifted from *V*_GS_ = 270 mV to lower gate voltages of *V*_GS_ = −3 mV. However, for the MW-PECVD G-FET, the shift was much smaller: from 510 mV to 475 mV (see Figure 7b).

Subsequently, by filling the channel of the microfluidic system with higher spike S protein concentration, the shift of the Dirac point to the lower voltages was further observed. However, this shift becomes smaller with an increase in spike S protein concentration, and saturation is seen.

**Figure 8:**
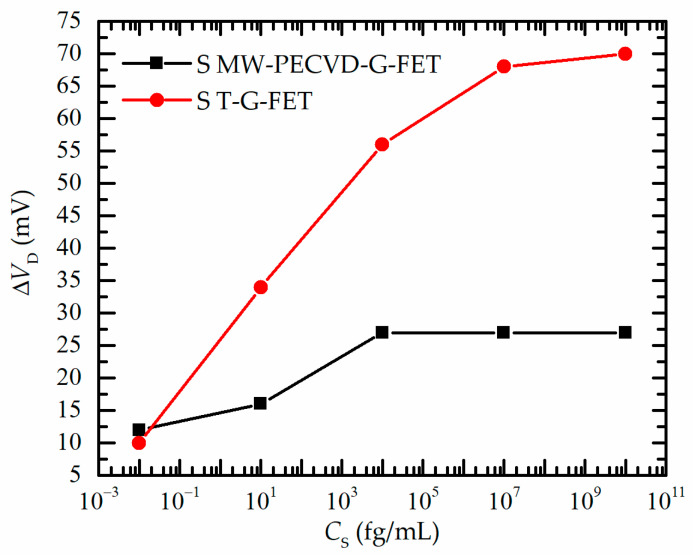
Change of *V*_D_ vs. spike S protein concentrations for PECVD and transferred graphene sensors.

Figure 8 summarizes the data presented in Figure 7. One can see that both G-FET devices showed remarkable sensitivity and were capable to detect the S spike protein with a concentration as low as 10^−2^ fg/mL. At this concentration, the Dirac voltage was shifted by ~10 mV. With an increase in the spike S protein concentration *C*_S_, the shift of the Dirac voltage ΔV_D_ increased with the tendency of saturation. However, for MW-PECVD-G-FET biosensors, the ΔV_D_ started saturating at a spike S concentration of 10^4^ fg/mL (shown as black squares in Figure 8), with the maximum Dirac voltage change being Δ*V*_D_ = 27 mV. On the other hand, the T-G-FET biosensor can operate across a broader range of the spike S concentrations, and the gate voltage shift tended to saturate only at 10^7^ fg/mL (depicted as red dots). In this case, the total shift of the Dirac voltage achieved approximately Δ*V*_D_ = 70 mV. From the analysis of the obtained results, it became evident that the characteristics and quality of pristine graphene have a significant influence on all modification processes of graphene surface, such as functionalization by PBASE, immobilization by ACE2, and S protein detection.

Furthermore, it should be highlighted that the detection of biomolecules using FET devices could be performed not only by measuring the change in the Dirac voltage. Other measurable parameters of the transfer curves that could change during the detection of analytes are the change in the transconductance *g*_m_ = d*I*_DS_/d*V*_GS_ (the slope of the transfer curves) and the relative change in current Δ*I*_DS_/*I*_0DS_ (or conductance) at a constant gate voltage, which is frequently measured at the CNP or in the p- or n- branches at a constant *V*_GS_. This method allows fast and easier measurement, which could be performed in real-time. It is crucial to carefully select the gate voltage at which the current exhibits the most significant change in the concentration of the analyzed biomolecules to achieve maximum sensitivity. By calculating the derivatives of the transfer curves shown in Figure 8, it was determined that for MW-PECVD-G-FET biosensors, the largest slope was at *V*_GS_ ≈ 200 mV, while for T-G-FET biosensors, it was ≈ −200 mV. Therefore, the relative change in current Δ*I*_DS_/*I*_0DS_ was calculated at these gate voltages. It was found Δ*I*_DS_/*I*_0DS_ ≈ 5.6% for the MW-PECVD-G-FET and ≈8.8% for T-G-FET for a concentration of spike S protein equal to *C*_S_ = 10 pg/mL and 6% and 10%, respectively, for *C*_S_ = 10 μg/mL. However, it is worth mentioning that this method has accuracy limitations because when biological analytes interact with the sensor, two distinct effects can occur. These effects are a shift of the Dirac point due to a change in the charge carrier density and a decrease in transconductance (slope of the transfer curve) in one or both branches of the characteristic due to an increase in charge carrier scattering [21]. Therefore, the measurement of the relative change of the current at a specific constant gate voltage is the simplest approach and could be used in real-time measurements. However, its interpretation becomes challenging, as it may involve contributions from both mentioned mechanisms, making it difficult to evaluate the individual contribution.

The obtained results demonstrate that although the MW-PECVD-grown graphene biosensor has a narrower range of sensitivity and a higher level of detection in comparison to the transferred graphene biosensor, it can be successfully used to detect SARS-CoV-2 spike S protein. MW-PECVD-G-FET can still sense tens of ag/mL concentration. Despite much larger defect density, a relative change in current Δ*I*_DS_/*I*_0DS_ because of the spike S protein action for directly grown graphene biosensor was comparable with the transferred graphene (5.6% and 8.8%, respectively), while MW-PECVD-G-FET sensitivity to the receptor ACE2 even exceeded the sensitivity of the T-G-FET. Considering these results and the compatibility of the graphene direct growth on the Si/SiO_2_ surface with Si technology, the directly synthesized graphene-based FET biosensor is very promising for integration into the lab-on-chip devices. Further optimization of the directly synthesized graphene structure is necessary to increase the sensitivity of the MW-PECVD-G-FET and their operating concentration range. It should be mentioned that the dependence of the directly synthesized graphene-based FET biosensor sensitivity on the structure remains unclear. Particularly, the graphene containing smaller grains and more grain boundaries exhibited lower average mobility [78]. However, the line defects and grain boundaries increased the sensitivity of the polycrystalline graphene-based chemoresistors [79]. Polycrystalline graphene-based resistor sensitivity to the adsorbed gas molecules was substantially higher than the sensitivity of the grain boundary-free graphene. However, the sensitivity depended on the grain boundary number, too [80]. Even more important seems to be the control of the substrate-induced self-doping of the graphene. A reduction in the substrate-induced self-doping resulted in both increased mobility of the transferred graphene [81,82] and increased sensitivity of the transferred graphene-based FET gas sensors [83]. The effects of substrate-induced doping can be reduced by passivation overlayer deposition [78]. However, this method is unsuitable for graphene-based biosensors because the passivation layer increases the distance between the graphene and analyte molecules and, as a result, reduces the sensitivity of the sensor. More perspective seems to be a selection of the appropriate substrate [81] and finding the optimal substrate pre-treatment before the graphene synthesis [84].

## 4. Conclusions

In conclusion, the graphene-based Field-Effect Transistor (G-FET) biosensors for the detection of SARS-CoV-2 spike S protein were developed. The G-FETs biosensors were fabricated using graphene directly synthesized on SiO_2_/Si substrate by MW-PECVD. Their sensitivity was comparable with the sensitivity of the FETs based on commercial CVD-grown graphene transferred on glass.

For the investigations of the detectivity and sensitivity of graphene biosensors, a microfluidic system with a liquid-gated FET circuit was developed. It was found that the functionalization of graphene by PBASE led to a shift of the Dirac voltage (*V*_D_) (charge neutrality point) to higher values, while immobilization by receptor ACE2 led to the shift of *V*_D_ to lower voltages. It can be explained by graphene p-doping and n-type doping effects, respectively. For G-FET with directly synthesized graphene, the PBASE adsorption-induced shift was significantly smaller, indicating the influence of the structure and properties of the pristine graphene layer. However, directly synthesized graphene FET sensitivity to the receptor ACE2, evaluated in terms of the Dirac voltage shift, exceeded the sensitivity of the transferred commercial graphene-based FET. Moreover, the shift in the Dirac voltage upon ACE2 immobilization could be attributed to charge transfer and changes in the doping level of the graphene.

Testing of the fabricated sensors for detecting SARS-CoV-2 spike S protein demonstrated that both types of G-FETs exhibited remarkable sensitivity and low detection limit and could detect S spike even at concentrations as low as 10^−2^ fg/mL. However, directly synthesized graphene exhibited slightly lower sensitivity compared to transferred graphene but sufficient for detection at low concentrations. Moreover, MW-PECVD G-FET biosensor showed saturation tendency at 10^4^ fg/mL, while T-G-FET based on transferred graphene could detect up to 10^7^ fg/mL spike S protein. The sensitivity estimation was also performed at a constant gate voltage corresponding to the largest transconductance values of both G-FETs. The relative source-drain current change for S protein concentration of 10 pg/mL was found to be 5.6% and 8.8% for directly synthesized and transferred graphene biosensors, respectively. Thus, it was comparable for both FET-based biosensors, despite the much larger defect density and nanocrystalline nature of the directly synthesized graphene.

The choice of graphene type, the structural quality of the graphene, and the functionalization process significantly affected the sensitivity and detection range of the G-FET biosensors. Overall, the results indicate that graphene-based G-FET biosensors have promising potential for sensitive detection of SARS-CoV-2 spike S protein, with further optimization of graphene quality and device fabrication, potentially enhancing their performance.

## Figures and Tables

**Table 1:** Graphene direct synthesis conditions.

Plasma Power (kW)	H_2_ Gas Flow (sccm)	CH_4_ Gas Flow (sccm)	Pressure (mBar)	Temperature (°C)	Time (min)
0.7	75	25	10	700	120

**Table 2:** Graphene Raman scattering spectra parameters.

Graphene Type	I_D_/I_G_	I_2D_/I_G_	I_D_/I_D’_	Pos(G) (cm^−1^)	Pos(2D) (cm^−1^)	FWHM(G) (cm^−1^)	FWHM(2D) (cm^−1^)
Directly synthesized graphene	1.46	0.42	3.26	1595.8	2703.3	37.6	75
Transferred graphene	0.18	2.05	0.55	1586	2685.6	15.7	32.8

## Data Availability

Not applicable.
